# Impairment of platelet mitochondrial respiration in patients with chronic kidney disease with and without diabetes

**DOI:** 10.1007/s11010-025-05280-5

**Published:** 2025-04-12

**Authors:** Glăvan Mihaela-Roxana, Stanciu-Lelcu Theia, Aburel Oana-Maria, Bînă Anca-Mihaela, Avram Vlad-Florian, Balint Lavinia, Gădălean Florica, Vlad Adrian, Sturza Adrian, Petrica Ligia, Muntean Mirela-Danina

**Affiliations:** 1 Department of Internal Medicine II – University Clinic of Nephrology, “Victor Babeș” University of Medicine and Pharmacy of Timișoara, Timișoara, Romania; 2 Centre for Molecular Research in Nephrology and Vascular Disease, “Victor Babeș” University of Medicine and Pharmacy of Timișoara Romania, Timișoara, Romania; 3 Department of Functional Sciences – Chair of Pathophysiology, “Victor Babeș” University of Medicine and Pharmacy of Timișoara, Timișoara, Romania; 4 Centre for Translational Research and Systems Medicine, “Victor Babeș” University of Medicine and Pharmacy of Timișoara, Timișoara, Romania; 5 Department of Internal Medicine II – University Clinic of Internal Medicine, Diabetes, Nutrition and Metabolic Diseases, “Victor Babeș” University of Medicine and Pharmacy of Timișoara, Timișoara, Romania

**Keywords:** Albuminuria, Chronic kidney disease, Diabetic kidney disease, Platelet mitochondrial respiration

## Abstract

Chronic kidney disease (CKD) and diabetic kidney disease (DKD) are major public health problems, and their burden is growing relentlessly with the aging of the global population. Their early recognition is now a public health priority, and there is an unmet need for the identification of specific biomarkers in minimally invasive or non-invasive samples. Mitochondrial dysfunction plays a pivotal role in the development and progression of both CKD and DKD and circulating platelets have emerged as an ideal candidate for the assessment of the respiratory function. The present study assessed mitochondrial respiration in platelets isolated from the peripheral blood of patients with DKD and CKD compared to healthy controls. The study included a total number of 89 subjects, as follows: 30 DKD patients divided into three subgroups based on the urinary albumin-to-creatinine ratio (uACR): 20 normoalbuminuric, 10 microalbuminuric, and 10 macroalbuminuric, 29 CKD patients (positive controls) and 20 healthy individuals (negative controls). Platelets were isolated by differential centrifugations and a high-resolution respirometry protocol was adapted to assess mitochondrial respiration dependent on complex I (CI) and complex II (CII). A significant reduction of the CI-supported active respiration was found in the normoalbuminuric DKD patients and further decreased in the microalbuminuric DKD subgroup. Both CI and CII-dependent coupled respiration and the maximal uncoupled respiration were significantly reduced in the macroalbuminuric DKD subgroup. In conclusion, mitochondrial respiration impairment in peripheral platelets is evident from the early stages of DKD. Moreover, platelet mitochondrial respiration was more severely impaired in patients with macroalbuminuric DKD as compared to those with CKD. Further, more extensive follow-up studies are warranted to determine whether platelet respiratory mitochondrial dysfunction could serve as a peripheral biomarker for kidney mitochondrial dysfunction and/or as a prognostic tool in DKD.

## Introduction

Chronic kidney disease (CKD), the end-stage of all chronic renal diseases and a complication of other chronic pathologies, in particular diabetes mellitus (DM) and hypertension, is defined as a decrease in the estimated glomerular filtration rate (eGFR) below 60 mL/min/1.73 m^2^ for at least 3 months and its stages are further classified based on both eGFR and albuminuria categories [1]. Currently, CKD represents a significant public health problem since it involves about 10% of the global population, and its burden is growing relentlessly with the aging of the population [2] with 85% of the cases being diagnosed in low- and middle-income countries [3]. Moreover, a recent report of the International Society of Nephrology found the highest median CKD prevalence (12.8%) in Eastern and Central Europe [2].

The age-related increase in CKD is primarily attributed to the increased prevalence of interrelated comorbidities, such as obesity, diabetes, and cardiovascular disease, and has led to the recent conceptualization of the so-called cardiovascular-kidney-metabolic syndrome view an early diagnostic of the disease (i.e., before the eGFR decrease) [4].

Diabetic kidney disease (DKD) is the most common microvascular complication of DM that occurs in approx. 40% of the patients and is the leading cause of CKD and progression toward end-stage renal disease (ESRD) [5]. While microalbuminuria is an early marker of DKD used as a routine for screening, several epidemiological studies have reported that DKD progression toward ESRD may occur through two distinct pathways, albuminuric and non-albuminuric (with the latter becoming increasingly prevalent) [6].

A very recent consensus statement published by three important societies, International Society of Nephrology, European Renal Association and American Society of Nephrology advocated for the inclusion of CKD on the list of major non-communicable diseases and postulated as public health priorities, acceleration of the knowledge and implementation of sustainable solutions for its early detection [7]. As such, identification of specific, cost-effective biomarkers in minimally invasive or non-invasive samples for early diagnostic purposes represents an unmet priority for both CKD and DKD.

Mitochondrial dysfunction has gained recognition in the pathophysiology of acquired kidney diseases more than one decade ago [8, 9] and has been reported to mechanistically encompass all the mitochondrial functions, among which alterations of biogenesis, redox homeostasis, dynamics, and bioenergetics have been mostly investigated [10]. More importantly, mitochondrial dysfunction occurs early in the evolution of DKD since it has been demonstrated that abnormalities in mitochondrial bioenergetics and dynamics precede the development of glomerular and tubular damage in experimental diabetes [11].

Mitochondrial oxidative phosphorylation (OXPHOS) is crucial for maintaining cellular energy homeostasis; however, assessing organ mitochondrial bioenergetic dysfunction in humans with renal diseases can be challenging as it requires an invasive kidney biopsy.

Circulating platelets and leucocytes (peripheral blood mononuclear cells, PBMCs) are nowadays used as an alternative to tissue biopsies for the study of mitochondrial bioenergetics [12]. Moreover, it has been recently suggested that the severe impairment of mitochondrial respiration can be detected more accurately in platelets as compared to PBMCs [13]. Platelets represent a population of easily available cells whose metabolic phenotype consists of a high coupling efficiency to mitochondrial ATP generation; as such, they have been increasingly used lately as liquid biopsies of mitochondrial respiratory dysfunction in the setting of numerous acute and chronic pathologies [14].

Platelet mitochondrial respiratory dysfunction has been reported to occur in patients with type 2 DM in a pioneering work published more than a decade ago [15]. However, whether the changes in platelet respiration are related to the severity of disease complications, such as DKD, has not been investigated to date.

The present pilot study aimed to assess mitochondrial respiratory function in peripheral platelets isolated from diabetic patients with DKD with and without albuminuria, compared with non-diabetic CKD patients and healthy controls.

## Materials and methods

### Study groups

The study was performed in accordance with the Declaration of Helsinki. Patient enrollment commenced after the study protocol was approved by the Ethics Committee of “Victor Babeș” University of Medicine and Pharmacy of Timișoara (No. 54/09.11.2020) and the Ethics Committee of “Pius Brînzeu” Emergency Hospital of Timiș County (No. 222/04.02.2021), respectively. All participants were informed regarding the experimental protocol, and written informed consent was obtained before participation.

Thirty patients with type 2 DM were recruited from the University Clinic of Nephrology and the University Clinic of Internal Medicine—Diabetes, Nutrition, and Metabolic Diseases from “Pius Brînzeu” Emergency Hospital from Timișoara, Romania. The DKD group was further divided into three subgroups according severity of the renal impairment, i.e., the urinary albumin/creatinine ratio (uACR), as follows: A1—normoalbuminuria: uACR < 30 mg/g (n = 20); A2—microalbuminuria: uACR 30—300 mg/g (n = 10); A3—macroalbuminuria: uACR ≥ 300 mg/g (*n* = 10). The second group included 29 patients with non-diabetic CKD (defined and staged according to the KDIGO Guideline for the Diagnosis and Management of CKD [1], which served as a positive controls. Patients with CKD were further subdivided according to the eGFR into two subgroups: P1 (early disease, *n* = 11) – eGFR > 60 ml/min/1.73 m^2^ and P2 (advanced disease, *n* = 18) – eGFR ≤ 60 ml/min/1.73 m^2^. Additionally, 20 age-matched individuals with no diabetes or CKD were recruited from the general physicians’ records and included in the third group, serving as the negative controls.

The exclusion criteria in both groups of patients with kidney disease were: the requirement of renal replacement therapies, sepsis, pregnancy, and neoplasia. All patients in the DKD group were receiving treatment with angiotensin-converting enzyme inhibitors or angiotensin II receptor blockers, oral antidiabetic medications and/or insulin, and statins. Patients in the non-diabetic CKD group were under treatment with angiotensin II converting enzyme inhibitors or angiotensin II receptor blockers, and most of them were also receiving statin therapy.

Clinical and laboratory data were collected and are presented in Table 1 as means and standard deviation (SD).

**Table 1:** Demographic, clinical, and biological characteristics of the participants

	Control group	Non-diabetic CKD group	DKD group		
			A1	A2	A3
Number of subjects	20	29	20	10	10
Clinical characteristics					
Age (y)	55.85 ± 9.30^*^	56.62 ± 17.19	69 ± 4.28	69.5 ± 4.25	68.5 ± 3.77
Female sex (no., %)	8 (40%)	21 (72.4%)	9 (45%)	3 (30%)	6 (60%)
CKD duration (y)	0	4.69 ± 3.49	1.55 ± 1.82	4.6 ± 3.09	4.2 ± 3.19
DM duration (y)	0	0	14.45 ± 6.67	17.4 ± 8.03	17.8 ± 5.63
Hypertension duration (y)	0	7.69 ± 6.23	5 ± 5.43	6.8 ± 3.80	9 ± 5.18
Glomerulonephritis (no., %)	0	9 (31%)	0	0	0
Laboratory parameters					
eGFR (mL/min/1.73 m^2^)	84.09 ± 4.56	46.86 ± 27.7*	59.83 ± 6.94*	56.65 ± 5.6*	50.14 ± 9.99*
uACR (mg/g)	11.91 ± 3.12	1252 ± 1690*	18.49 ± 7.113	86.27 ± 57.318*	688.3 ± 482.2*
HbA1c (%)	4.97 ± 0.23	5.15 ± 0.31	7.12 ± 0.98*	7.71 ± 1.17*	8.73 ± 0.97*

A1-normoalbuminuria (uACR < 30 mg/g); A2-microalbuminuria (uACR 30–300 mg/g); A3-macroalbuminuria (uACR ≥ 300 mg/g); CKD-chronic kidney disease; DKD-diabetic kidney disease; DM-diabetes mellitus; eGFR-estimated glomerular filtration rate; uACR-urinary albumin/creatinine ratio; HbA1c-hemoglobin A1c. Data are presented as mean ± SD *vs* the control group.

### Assessment of platelet respiration by high-resolution respirometry

#### Platelet isolation

Platelet isolation was performed in 1–4 h after blood sampling according to a protocol described in ref. [16]. In brief, venous blood (20 mL) was drawn in K_2_EDTA tubes. Two consecutive centrifugations were performed at room temperature, as follows: the first centrifugation of the fresh blood at 500×*g* for 10 min to obtain the platelet-rich plasma and the second centrifugation at 4600×*g* for 5–10 min to obtain the platelet pellet. The pellet was resuspended in autologous plasma (approx. 1 mL) and further used for the high-resolution respirometry (HRR) experiments. Platelet count was performed using an automatic cell counter (Swelab Alfa Plus, Sweden).

#### Assessment of platelet mitochondrial respiration by means of HRR

Assessment of mitochondrial respiratory function in isolated platelets was conducted at 37 °C using the oxygraph-O2k (Oroboros Instr., GmbH Innsbruck, Austria). The classic HRR protocol Substrate-Uncoupler-Inhibitor-Titration (SUIT) was adapted to measure respiration supported by complexes I and II of the electron transport system. Platelets were suspended in a concentration of 200 × 10^6^ cells/mL in the 2 ml glass chambers of the oxygraph containing the MiR05 respiratory solution (composition: EGTA 0.5 mM, potassium lactobionate 60 mM, MgCl_2_^**.**^6H_2_O 3 mM, KH_2_PO_4_ 10 mM, sucrose 110 mM, taurine 20 mM, HEPES 20 mM, and 1 g/L bovine serum albumin, pH adjusted at 7.1).

Platelet respiration was evaluated according to previously described protocol [16].

In brief, platelets were initially allowed to be stabilize for approx. 15 min, thus allowing the assessment of ROUTINE respiration (respiration in the presence of endogenous substrates). Subsequently, platelets were permeabilized by the addition of digitonin (1 µg/L × 10^6^ platelets) in order to enable the mitochondrial entry of the exogenous respiratory substrates and ADP. The measurement of complex I (CI)-dependent active respiration or OXPHOS CI was performed after the addition of the CI-substrates (glutamate 5 mM and malate 5 mM) and ADP (1 mM). An additional titration of succinate was administered to stimulate the maximum capacity of oxidative phosphorylation across both C me and II complexes or OXPHOS CI + CII. The evaluation of LEAK respiration (i.e., the respiration independent of ADP phosphorylation) was assessed after the addition of the ATP synthase inhibitor, oligomycin (1 μg/mL). By successive titrations of an uncoupler agent (that eliminates the proton gradient), FCCP (carbonyl cyanide p-trifluoromethoxy-phenylhydrazone), the maximal uncoupled respiration of the electron transport (ET) system or the ET CAPACITY was estimated. Finally, the non-mitochondrial respiration was evaluated by inhibiting complex I with rotenone (2 μM) and complex III with antimycin A (1 μg/mL), respectively. The resulting slow rates of O_2_ consumption known as the residual oxygen consumption (ROX), due to processes other than oxidative phosphorylation, were subtracted from the other respiratory rates.

DatLab software (Oroboros Instr. v.7.4) was used to record in real time the oxygen consumption rate and further for data analysis. Before each experiment calibration at air saturation was conducted and mitochondrial respiration was also corrected for oxygen flux (due to instrumental background).

During HRR experiments, the following respiratory parameters were measured: (i) ROUTINE respiration (basal respiration), (ii) OXPHOS capacity (maximal phosphorylating respiration), (iii) LEAK (non-phosphorylating respiration), and (iv) ET capacity (maximal uncoupled respiration) as previously published [16, 17].

### Chemicals

All reagents were purchased from Merck-Sigma-Aldrich.

### Statistical analysis

Data are presented as means ± SEM. Before conducting ANOVA analysis, assumptions regarding data distribution and homogeneity of variances were carefully verified. Data normality was evaluated using the Shapiro–Wilk test, and the homogeneity of variance across groups was assessed using Levene’s test. Statistical analyses and assumption checks were performed using GraphPad Prism software (v. 10.2.2; GraphPad Software, San Diego, CA, USA). Both normality and variance homogeneity criteria were met. For the group comparison, one-way ANOVA with Bonferroni’s posthoc test was performed. Significant differences are denoted: *p < 0.05; ** p < 0.01; *** p < 0.001, ****p < 0.0001.

## Results

### Platelet mitochondrial respiration is early impaired in DKD patients regardless the presence of albuminuria

**Fig. 1:**
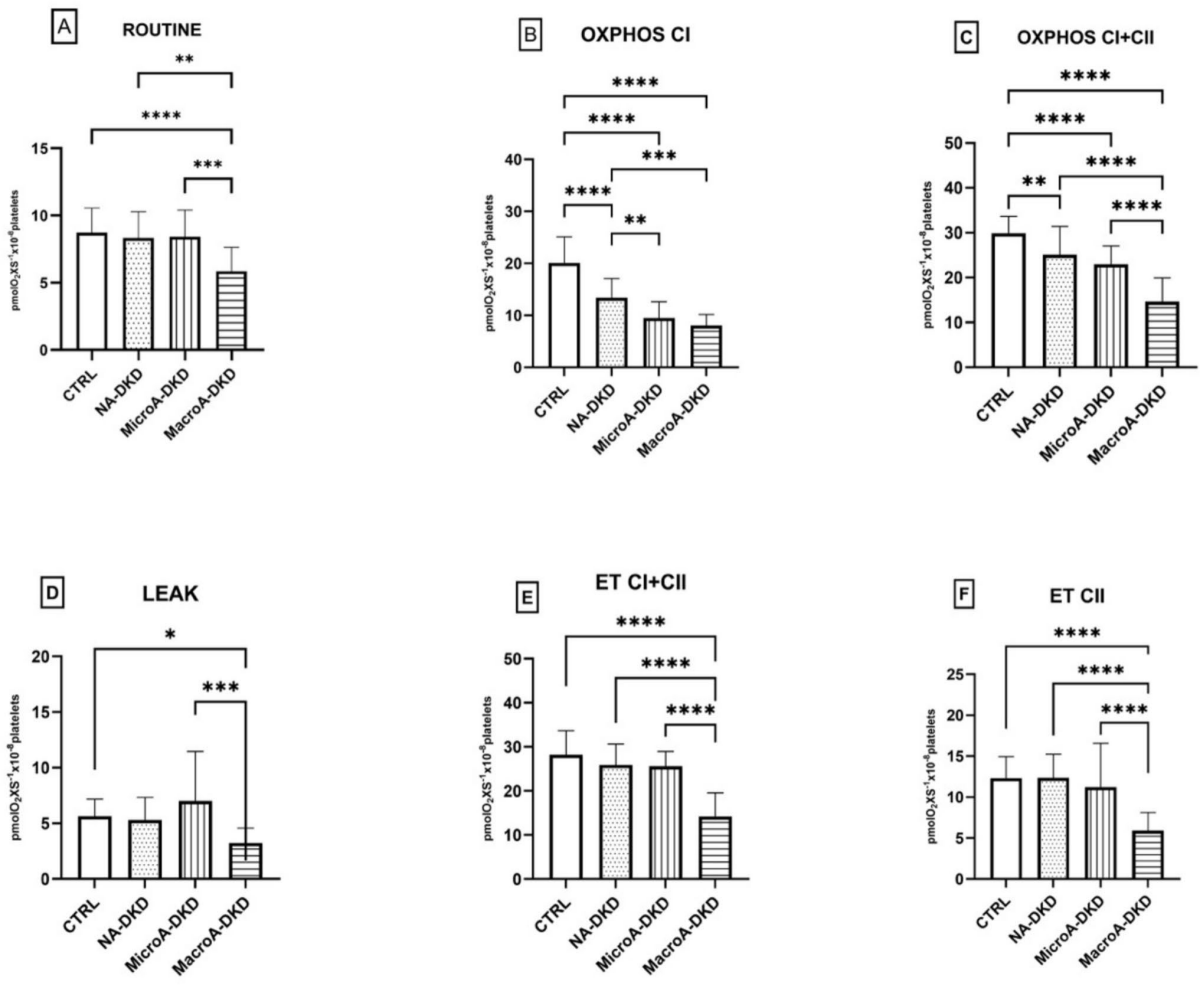
Respiratory parameters measured in platelets isolated from DKD patients. *CTRL* control group, *NA-DKD* normoalbuminuric diabetic kidney disease, *MicroA-DKD* microalbuminuric diabetic kidney disease, *MacroA-DKD* macroalbuminuric diabetic kidney disease, *ET* electron transport, *OXPHOS* oxidative phosphorylation. One-way ANOVA with Bonferroni’s post hoc test was performed. *p < 0.05; **p < 0.01; ***p < 0.001; ****p < 0.0001

Platelet mitochondrial respiratory parameters were assessed in the DKD group and its corresponding subgroups (A1, A2, and A3) and compared with healthy subjects. As shown in Fig. 1A, basal respiration (ROUTINE) did not differ between the normoalbuminuric A1 and microalbuminuric subgroup A2, and the healthy controls; only in the macroalbuminuric A3 group ROUTINE respiration was significantly decreased (*p* < 0.0001). At variance, both the OXPHOS capacity for CI and OXPHOS CI + CII (the maximal phosphorylating capacity) were significantly lowered already from the normoalbuminuric A1 stage and further decreased in the following two stages. Interestingly, when compared with controls, the OXPHOS decrease in the A1 group was more important for CI (*p* < 0.0001, Fig. 1B) *vs* the CI + CII-supported active respiration (*p* < 0.01, Fig. 1C), pointing to an early impairment of the complex I of the ET system in CKD patients lacking albuminuria. This difference was preserved for the patients with microalbuminuria, while in the macroalbuminuric patients the active ATP generating respiration displayed the most severe decrease for both complexes (*p* < 0.0001, Fig. 1B and C) as compared to the healthy subjects.

LEAK (or the non-ATP–generating respiration) was non-affected in the normoalbuminuric A1 DKD subgroup and significantly decreased in the macroalbuminuric (A3) subgroup *vs* controls (*p* < 0.05) – Fig. 1D.

The ET capacity, a parameter that assesses the maximal activity of the electron transport system (by measuring oxygen consumption in the presence of an optimal concentration of the FCCP protonophore), was significantly decreased only in the A3-DKD subgroup as compared to the controls (*p* < 0.0001). Both ET I + II and ET II showed comparable, non-altered values between the healthy subjects and the A1 and A2 subgroups, respectively (Fig. 1E and F).

### Platelet mitochondrial respiration is significantly impaired in non-diabetic chronic kidney disease

In a distinct series of experiments, we measured respiratory parameters in platelets harvested from non-diabetic patients with CKD as compared to healthy controls.

**Fig. 2:**
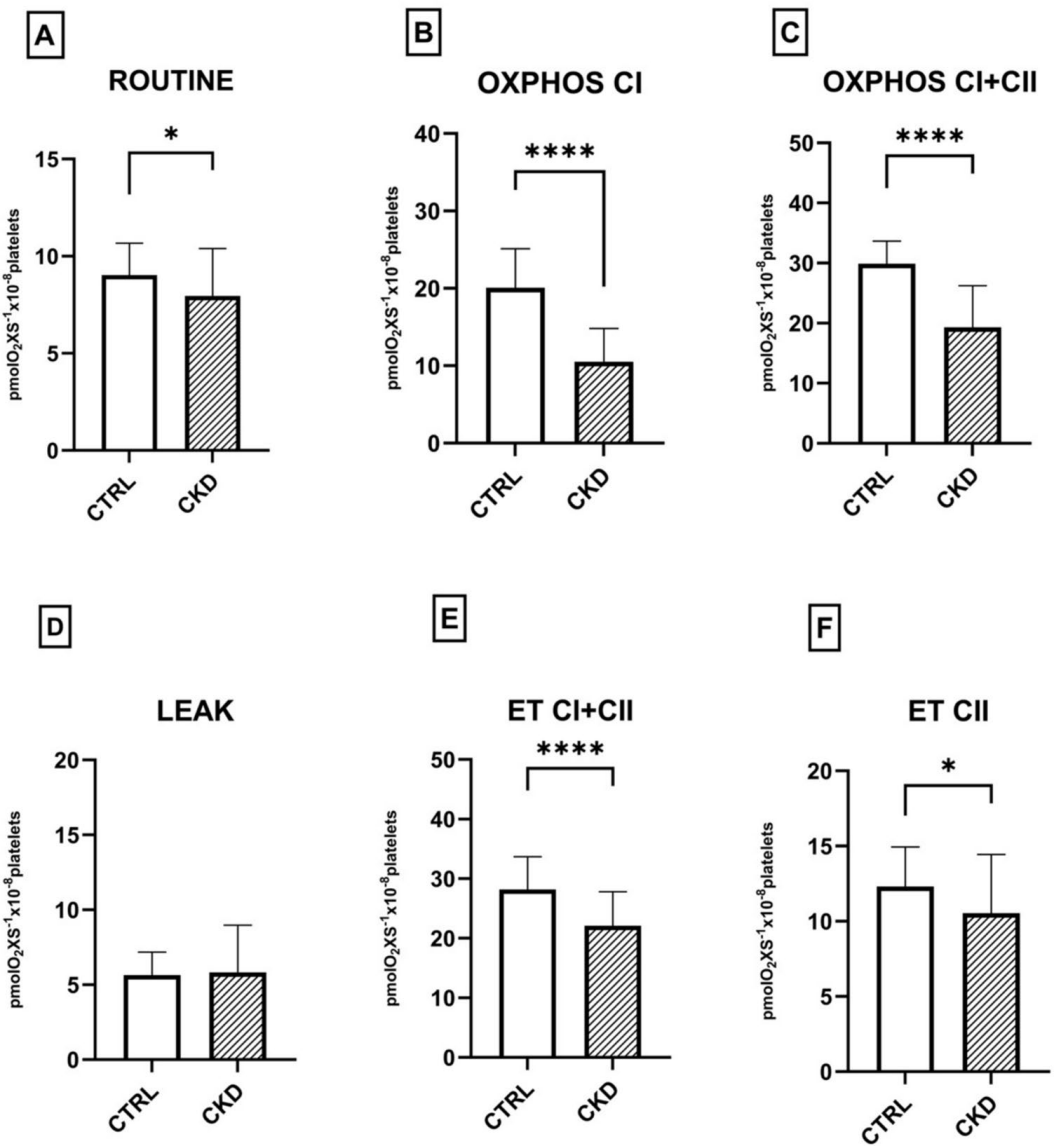
Respiratory parameters measured in platelets isolated from non-diabetic CKD patients. *CTRL* control group, *CKD* chronic kidney disease, *OXPHOS* oxidative phosphorylation, *ET* electron transport. One-way ANOVA with Bonferroni’s post hoc test was performed. *p < 0.05; ** *p* < 0.01; *** *p* < 0.001; **** *p* < 0.0001

As depicted in Fig. 2, all respiratory parameters (except for LEAK) were significantly reduced in patients with CKD. Both OXPHOS and ET capacity displayed low values for CI and CII (*p* < 0.0001), indicating a significant impairment of platelet mitochondrial respiration in CKD patients, which results in reduced ADP phosphorylation capacity.

Patients in this group were further divided into two subgroups according to the eGFR: early CKD (P1, eGFR > 60 mL/min/1.73 m^2^) and advanced CKD (P2, eGFR ≤ 60 mL/min/1.73 m^2^) and respiratory parameters compared between these subgroups versus controls. Interestingly, we observed that ET CII capacity was decreased in group P2 compared with group P1 but without reaching statistical significance.

**Fig. 3:**
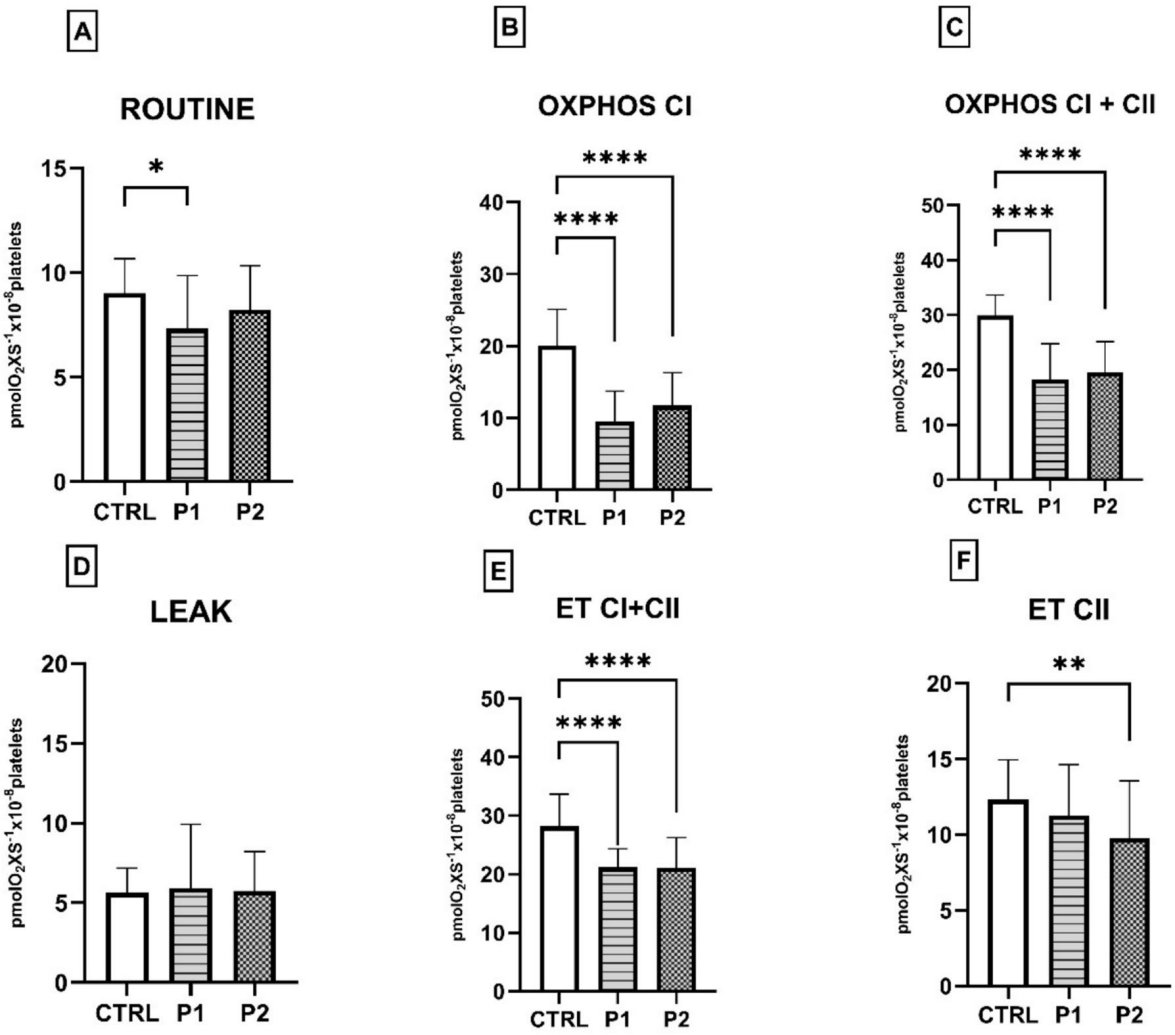
Respiratory parameters measured in platelets isolated from CKD subgroups according to the eGFR. *CTRL* control group, *P1* early CKD (eGFR > 60 mL/min/1.73 m^2^), *P2* late CKD (eGFR ≤ 60 mL/min/1.73 m.^2^), *OXPHOS* oxidative phosphorylation, *ET* electron transport. One-way ANOVA with Bonferroni’s post hoc test was performed. *p < 0.05; **p < 0.01; ***p < 0.001; ****p < 0.0001

As depicted in Fig. 3, despite a significant reduction in both coupled and uncoupled platelet respiration versus the control group, no significant differences were found between early and late CKD. The observation points to the fact that this peripheral biomarker is not sensitive enough to detect stage-related impairment in mitochondrial function in the present pilot study.

### Platelet mitochondrial respiration is more severely impaired in patients with macroalbuminuric DKD as compared to non-diabetic CKD

**Fig. 4:**
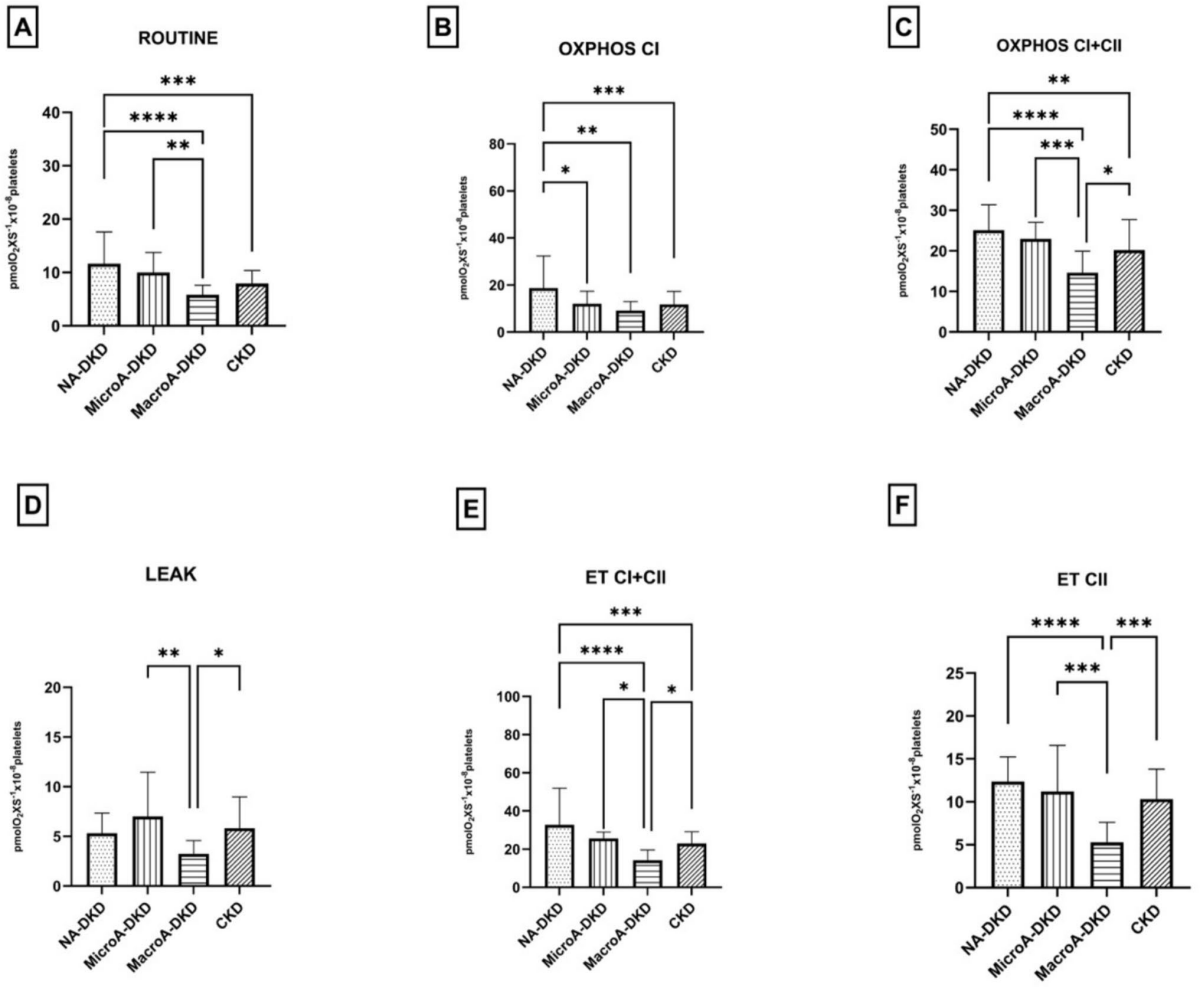
Respiratory Parameters in Platelets Isolated from DKD *vs* CKD patients. *CTRL* control group, *NA-DKD* normoalbuminuric diabetic kidney disease, *MicroA-DKD* microalbuminuric diabetic kidney disease, *MacroA-DKD* macroalbuminuric diabetic kidney disease, *ET* electron transport, *OXPHOS* oxidative phosphorylation. One-way ANOVA with Bonferroni’s post hoc test was performed. *p < 0.05; **p < 0.01; ***p < 0.001; ****p < 0.0001

Respiratory parameters from the subgroups of DKD (normo-, micro-, and macroalbuminuric) were compared with those recorded in the non-diabetic CKD group (Fig. 4). The most important finding is that patients in the macroalbuminuric A3 DKD subgroup exhibited lower values for both coupled and uncoupled respiration compared to the CKD group. As shown in Fig. 4, statistical significance (*p* < 0.05) was observed for OXPHOS CI + CII (Fig. 4C, *p* < 0.05), LEAK (Fig. 4D, *p* < 0.05), ET CI + CII (Fig. 4E, *p* < 0.05), and ET CII (Fig. 4F, *p* < 0.001), while the other parameters also displayed a decreasing trend.

## Discussion

Platelet hyperactivity and dysfunction in the setting of diabetes has been reported more than 2 decades ago when the research activity was tackling the abnormal hemostasis [18] and has been associated not only with hyperglycemia but also with the other conditions (insulin resistance, obesity, dyslipidemia) that contribute to chronic systemic inflammation and oxidative stress [19]. Also, CKD has been reported to impair hemostasis by inducing several platelet abnormalities [20] with both inhibitory and stimulatory effects [21].

In both CKD and DKD, the disruption of energy metabolism resulting from impaired renal oxygen levels has emerged as a major subcellular pathomechanism. In the setting of DM, it has been postulated that kidney mitochondrial dysfunction is a significant upstream consequence of prolonged hyperglycemia [10]. Alteration of the delivery of metabolic substrates results in increased oxygen consumption that contributes to renal hypoxia [22]. Also, there is unequivocal experimental evidence in the literature that impaired renal mitochondrial bioenergetics precede the classic ultrastructural changes (podocyte loss, basement membrane thickening, mesangial expansion, tubular atrophy, and interstitial inflammation/fibrosis) and contribute to the progression of DKD toward ESRD [23]. Whether the kidney mitochondrial dysfunction is translated in systemic impairment of bioenergetics has not been systematically addressed.

An increasing body of evidence supports the use of peripheral blood cells, mainly platelets (but also PBMCs), as biomarkers for mitochondrial functional health. As such, mitochondrial respiratory dysfunction in peripheral blood cells has been described in several acute and chronic pathologies, such as sepsis and sepsis induced neurological dysfunctions [24, 25], severe acute COVID- 19 infection [26], preeclampsia [16], acute hematological malignancies [27, 28], cardiopulmonary by-pass [29, 30], pulmonary embolism [31], trauma [32], carbon monoxide intoxication [33, 34], long term HIV infection [35], diabetes mellitus [15, 36], non-alcoholic fatty liver disease [37], depression [38, 39], Alzheimer’s disease [40, 41] and other pathologies (recently reviewed in ref. [12]).

Our findings also support the role of blood-based bioenergetic profiling as a minimally invasive approach for assessing systemic mitochondrial dysfunction. The major finding of this study is the decrease in platelet active respiration (OXPHOS CI + CII) in patients with normoalbuminuric DKD suggesting the occurrence of an early systemic mitochondrial dysfunction in the evolution of type 2 DM associated with the major microvascular complication, the diabetic kidney. A significant reduction mainly of the CI-supported active respiration was found already in the normoalbuminuric A1-DKD subgroup and further decreased in the microalbuminuric A2-DKD patients. Both CI and CII-dependent coupled respiration and also the maximal uncoupled respiration (ET CI + CII) were significantly decreased in the macroalbuminuric A3-DKD subgroup. In the latter group, LEAK respiration was also significantly decreased.

In patients with CKD, all respiratory parameters (except LEAK respiration) were significantly lower compared to healthy controls. This finding suggests the potential use of platelet respiration impairment as a peripheral marker of bioenergetic dysfunction in patients with CKD. Similarly, Altintas et al. compared the bioenergetic profiles of PBMCs harvested from patients previously diagnosed with end-stage chronic kidney disease (CKD) and healthy donors, revealing a significant decrease in basal, oxidative phosphorylation (OXPHOS), and maximal uncoupled respiration [42], which highlights the role of peripheral blood cells as systemic biomarkers of impaired mitochondrial bioenergetics.

When comparing the respiratory parameters between DKD and CKD, we showed that patients in the macroalbuminuric A3 DKD subgroup had lower values for both coupled and uncoupled respiration as compared to the CKD group. This observation indirectly suggests the more severe impairment of systemic bioenergetics in diabetic *vs* non-diabetic patients with chronic renal disease. The group of Michael Sacks firstly reported that platelets isolated from patients with type 2 DM exhibited a reduced oxygen consumption and ATP synthesis as compared to those isolated from insulin-sensitive controls [15]. More recently, Wang et al. performed an elegant study to characterize the bioenergetic profile of platelets from patients with type 2 DM who developed coronary in-stent restenosis (ISR) as compared with platelets from diabetics without ISR (non-ISR) and healthy control donors (HC). They reported the impairment of mitochondrial respiration with the decreased contribution of OXPHOS to ATP production and a compensatory enhancement of glycolysis in ISR platelets compared with non-ISR and HC platelets. They also found a high dependency of ISR platelets on fatty acid oxidation (FAO) associated with impaired respiration driven by complex III and increased ROS generation. Interestingly, inhibition of FAO with trimetazidine improved mitochondrial function and normalised ISR platelet activity, while activation of FAO with C75, a stimulator of carnitine palmitoyltransferase 1 in non-ISR platelets, mimicked mitochondrial dysfunction and platelet hyperreactivity [36]. At variance from these results, in a pilot study of 10 diabetic patients with CKD and arterial hypertension Gvozdjáková et al. reported no significant changes in platelet respiratory parameters as compared to controls [42].

Respiratory mitochondrial dysfunction in DKD has been also reported to occur in PBMCs. Czajka et al. assessed mitochondrial respiration in PBMCs harvested from type 1 and 2 diabetic patients with and without DKD *vs* healthy donors and reported a significant decrease in maximal respiration and reserve capacity (measured as the difference between maximal and basal respiration) in the setting of DKD [43].

Collectively, platelets are valuable samples for detecting real-time changes in mitochondrial bioenergetics driven by chronic renal disease in patients with and without diabetes.

### Study limitations

We must acknowledge several limitations of the present pilot study. Firstly, the relatively small sample size has a limited statistical power to detect subtle differences among the subgroups according to the disease severity (that cannot be excluded). Second, the heterogeneous etiology of CKD, albeit dominated by glomerulonephritis and hypertensive nephrosclerosis, elicits different degrees of glomerular mitochondrial dysfunction that variably impacts platelet respiration. Additionally, the effects of comorbidities and medication on mitochondrial respiration, as potential confounding factors, were not taken in account. Future research in larger cohorts, detailed assessment of confounding variables, and longitudinal studies would help address these limitations.

## Conclusions

In conclusion, we firstly demonstrated that platelet mitochondrial respiration is already altered in patients with normoalbuminuric DKD. Moreover, in patients with macroalbuminuric DKD, the impairment was more severe compared to those with CKD. Further studies are required to confirm whether assessing platelet respiration might have potential clinical applicability as a liquid biopsy for kidney mitochondrial dysfunction and/or as a prognostic tool in DKD.

## Data Availability

Data is provided within the manuscript.
